# Ethnoveterinary medicines used for horses in Trinidad and in British Columbia, Canada

**DOI:** 10.1186/1746-4269-2-31

**Published:** 2006-08-07

**Authors:** Cheryl Lans, Nancy Turner, Gerhard Brauer, Grant Lourenco, Karla Georges

**Affiliations:** 1 University of Victoria, Environmental Science, British Columbia, V8W 3P5, Canada; 2 University of Victoria, Health Information Science, British Columbia, V8W 3P5, Canada; 3 c/o Trinidad and Tobago Racing Authority, Santa Rosa Park, Churchill Roosevelt Highway, O'Meara, Arima, Trinidad and Tobago; 4 School of Veterinary Medicine, Faculty of Medical Sciences, University of the West Indies, Mt. Hope, Trinidad and Tobago

## Abstract

This paper investigates the commonalities in ethnoveterinary medicine used for horses between Trinidad (West Indies) and British Columbia (Canada). These research areas are part of a common market in pharmaceuticals and are both involved in the North American racing circuit. There has been very little research conducted on medicinal plants used for horses although their use is widespread. The data on ethnoveterinary medicines used for horses was obtained through key informant interviews with horse owners, trainers, breeders, jockeys, grooms and animal care specialists in two research areas: Trinidad and British Columbia (BC). A participatory validation workshop was held in BC. An extensive literature review and botanical identification of the plants was also done. In all, 20 plants were found to be used in treating racehorses in Trinidad and 97 in BC. Of these the most-evidently effective plants 19 of the plants used in Trinidad and 66 of those used in BC are described and evaluated in this paper. *Aloe vera*, *Curcuma longa *and *Ricinus communis *are used in both research areas. More research is needed in Trinidad to identify plants that respondents claimed were used in the past. Far more studies have been conducted on the temperate and Chinese medicinal plants used in BC and therefore these ethnoveterinary remedies reflect stronger evidence of efficacy.

## Background

Trinidad and Tobago is located northeast of the Venezuelan coast and has a humid tropical climate. British Columbia (BC) is the western-most province in Canada and has a temperate climate. This paper describes a selection of the ethnoveterinary medicines used for horses in Trinidad and Tobago and in British Columbia. These places are part of a common market in pharmaceuticals and are both involved in the North American horse racing circuit. Since racehorses and jockeys are often in transition from other regions and between Canada (including Woodbine racetrack in Ontario, the Aqueduct racetrack and Belmont Park, both in New York) and the Caribbean, one of the goals of this research was to investigate commonalities in ethnoveterinary medicine between these two regions. Very little research has been conducted on ethnoveterinary medicine used for horses and there are few comparative studies. There are some shared cultural features between Canada and the Caribbean derived from common Amerindian culture, British colonial histories, and substantial and continuous migration from the Caribbean to North America. An estimated 150,000 Trinidadians are currently living in Canada.

The population of Trinidad, just over 1 million people has equal proportions of African-origin and East Indian-origin (39%). Approximately 15% of the population consists of mixed raced persons and the remainder consists of minority groups (>2%) of European-origin, Middle-Eastern-origin and Chinese-origin people. British Columbia has a total population of 4.168 million people. The 1996 census revealed that 50% of the population was of European origin and 27% of Asian origin. The population of Chinese origin is estimated at 253,382. The 2001 Census revealed that the top 10 languages spoken in BC are: English, Chinese (including Cantonese and Mandarin), Punjabi, then five Western European languages, Tagalog and Korean.

There are major differences in vegetation between the two areas. However a few studies have revealed that geographical barriers are lessening in terms of increasingly globalized ethnomedicine. For example, one study conducted south of Trinidad revealed that of 216 introduced plant species used by peoples in northern South America (Brazil, Colombia, Ecuador and Peru), 80% were of European, Mediterranean or Asian origin, 9% were of African origin and 8% were from the New World [1]. Another researcher found that 36% of the taxa used in the Atlantic forests of Bahia, Brazil for which origins could be established came from Africa, Asia and Europe [2]. The plant pharmacopoeia in South America is cultivated, exotic and opportunistic and sourced from home gardens, roadsides and secondary forest rather than indigenous species from the primary forests that were alien to the region's new settlers [2]. Canadians use a wealth of herbs of European origin. However research conducted by the first author in both countries indicates that there are far more herbs of Chinese origin being used in Canada than there are in Trinidad and Tobago [3,4]. We will return to this point later in the paper.

Horse racing has been established in Trinidad since 1828 [5]. There are occasional race days as well on the twin island of Tobago. The only utilised racetrack in Trinidad was moved from the capital city (Port of Spain), east to the refurbished venue at the Santa Rosa Complex (73 hectares) in Arima in 1993. Previously, all races were run on a clockwise turf track. However due to the influence of American-style racing, an anti-clockwise sand track surface circuit was laid. Race distances vary from 350 metres (for 2-year-olds) to 2000 meters. The Santa Rosa Complex hosts 40 race days annually. The Trinidad and Tobago Racing Authority is the body responsible for horse racing. There are several associations associated with horse racing: the Owners, the Stud Farm, the Bookmakers, Grooms and Trainers Associations, the Arima Race Club and the Tobago Race Club. The government Minister in charge of horse racing in 1999 claimed it was not economically viable and relied heavily on government financial support [6]. Creoles (locally born horses) from Trinidad also race in Puerto Rico, Barbados and Jamaica. In the 2004 Agricultural Census conducted by the Ministry of Planning and Development, the Ministry of Agriculture, Lands and Marine Resources and the Central Statistical Office, horses were not counted and therefore data on the horse industry is limited.

The research area in British Columbia consisted of the Lower Mainland, the Thompson/Okanagan region and south Vancouver Island. The racetrack situated in the research area is Hastings Park, in Vancouver, the largest city in the province. The 2001 Statistics Canada Census revealed that there were 53,366 horses and ponies living on 6,820 farms in BC. The horse industry in 2001 was primarily located in the Thompson Okanagan (25%), the Lower Mainland (20%), the Peace River (18%), and lastly 15% in the Cariboo region. A typical horse unit has seven mares on 10–70 acres. The horse racing industry includes between 9,000 and 10,000 horses, generates $198 million annually and creates 4,000 jobs; but horse racing constitutes only 18% of the horse economic sector [7]. Other parts of the sector include recreational and trail riding, competitions, companionship and other kinds of working animals. The total economic activity involving horses in BC contributed 771 million dollars.

### Data collection

Data collection in Trinidad took place in 2000, with further work conducted in 2003. Data collection in BC was carried out in 2003. The respondents were ethnically and demographically varied. A selection of both sets of ethnoveterinary remedies is evaluated in the discussion section of the paper using a non-experimental validation method. The Trinidad component of this study was derived from a larger research project on ethnoveterinary medicines used in Trinidad and Tobago [4]. This previous study revealed that the main outcome or synergy in folk medicine is that all the knowledge is available to all ethnic groups in a kind of 'melting pot' and that there are no rigid barriers preventing the spread of knowledge between the various ethnic groups. In order to gain access to the study population the authors worked through previously known individuals and from previously existing social networks in building a snowball sample and hence a network of interviewees [8]. The first contact relating to this study was a race-horse owner (#8 top earner for the period 1994 to 2000); she drove the first author to the initial visit to the racetrack and to the broodmare farm where her horses were kept. She also introduced the author to several of the trainers. When respondents in the horse racing industry were contacted subsequently it was discovered that they already knew about the research from the initial contact.

Interviews in Trinidad took place from July to September 2000 (CL) and in 2003 (KG). The interviews conducted in Trinidad in 2003 reassessed the initial data (a form of triangulation). The research was facilitated by community-based contacts and occupationally based contacts obtained from newspapers. This networking approach was necessary because there is no sampling frame of persons involved in ethnoveterinary medicine in Trinidad. It produced the desired purposive sample of key respondents.

Four visits were made to the sole racetrack; one of these was on a race day. One visit each was made to three of the six brood mare farms in Trinidad, located in North, East and Central Trinidad. At the racetrack, ten trainers and two assistant trainers were interviewed and one retired trainer was interviewed by phone (this sample is one-tenth of all trainers in Trinidad). The sample frame for choosing the trainers was obtained from the sports pages of the three daily newspapers and from the statistics kept at the University of the West Indies library. All of the interviews were unstructured and open-ended. One of the trainers was also a practising veterinarian. Seven of the ten trainers are recorded in the statistics kept on the "top 25" winners (1994–2000) (#3, #4, #6, #7, #9, #14, #18).

Of all of the trainers interviewed two used no ethnoveterinary medicines, 25% were active users while others reported past use in the 1970s or what they had observed others using. Four grooms were interviewed; they were current users of ethnoveterinary medicines. Six owners/breeders or their representatives were interviewed in 2000, two of them by phone. Four were ranked among the "top 25" in winnings (1994 – 1998) (#1, #7, #8, #12); only one used ethnoveterinary medicines. Three of the six veterinarians consistently working with horses were interviewed, two reported their knowledge of ethnoveterinary medicines, one was also a trainer as indicated above, the other a former jockey.

In 2003, four trainers were interviewed (one by phone). One was selected to confirm the previous data; two were interviewed in 2000, but independently selected in 2003; one was new. Additionally a groom, a stable lad, an assistant trainer, a jockey and a recently graduated veterinarian were interviewed.

Ethnoveterinary data for British Columbia was collected over a six-month period in 2003. All available literature about livestock farmers and the secondary literature on ethnomedicinal plants, folk medicine and related fields in British Columbia was reviewed.

A purposive sample of livestock farmers was necessary to target key informants with the knowledge sought. The sample size was 60. The sample was obtained from membership lists of organic farmers, horse breeders and trainers, horse stables, other specialists in alternative medicine and holistic veterinarians.

Interviewees comprised one naturopath, four horse breeders/trainers, two herbalists, one farmer and one headmistress with horses at her school (for girls). All of the respondents used herbal medicines for horses. Two visits were made to each farm or respondent, and to the Hastings racecourse in Vancouver. All of the interviews at the initial stage were open-ended and unstructured. A draft outline of the respondents' ethnoveterinary remedies was delivered and discussed at the second visit in order to confirm the information provided at the first interview. Medicinal plant voucher specimens were collected where possible and were identified and deposited in the University of Victoria herbarium (V).

The plant-based remedies were evaluated for safety and efficacy with a non-experimental method, prior to including them in the draft outline. Published sources such as journal articles and books and databases on pharmacology and ethnomedicine available on the Internet were searched to identify the plants' chemical compounds and clinically tested physiological effects. This data was incorporated with data on the reported folk uses, and their preparation and administration in North America and Europe. For each species or genus the ethnomedicinal uses in other countries are given; followed by a summary of chemical constituents, in addition to active compounds if known. This type of ethnopharmacological review and evaluation is based on previous work and the use of these methods in a previous research study has been published [4,9-11]. The non-experimental validation of the plants is presented in the discussion section of the paper.

### Validation workshop

Ten participants with experience in traditional human and ethnoveterinary medicine took part in a participatory five-day-long workshop at the University of Victoria (BC), in October, 2003. In the workshop the facilitator asked participants very specific questions in a supportive environment about the medicinal plants used. Each animal/livestock species was covered in a morning or afternoon session [4,11]. At the horse session the four participants (two horse trainers and two herbalists), introduced themselves and their work and were instructed on the participatory workshop method. The participants discussed the previously produced horse section of the data. There were two editorial assistants/facilitators in attendance. After the discussions, the horse section was edited. In addition, two herbalists in Port Alberni were visited by the ethnoveterinary consultant and the researcher (CL) and the edited horse data was discussed with them. One trainer with horses at the Hastings racecourse visited the researcher after the workshop and discussed the workshop-edited horse data with the researcher and the ethnoveterinary consultant.

### Non-experimental validation of ethnoveterinary remedies

The researcher and the ethnoveterinary consultant completed the non-experimental validation of the remedies in advance of the workshop. A low-cost, non-experimental method was used to evaluate the potential efficacy of the ethnoveterinary remedies [9-11]. This method consisted of:

• obtaining an accurate botanical identification of the herbal remedies reported;

• searching the pharmaceutical/pharmacological literature for the plant's identified chemical constituents in order to determine the known physiological effects of either the crude plant drug, related species, or isolated chemical compounds that the plant is known to contain. This information was then used to assess whether the plant use is based on empirically verifiable principles.

Supporting ethnobotanical data and pharmacological information was matched with the recorded folk use of the plant species [12-18], to determine degrees of confidence about its effectiveness. Four levels of confidence were established:

1. Minimal level: If no information supports the use it indicates that the plant may be inactive.

2. Low level: A plant (or closely related species of the same genus), which is used in distinct areas in the treatment of similar illnesses (humans or preferably animals), attains the lowest level of validity, if no further phytochemical or pharmacological information validates the popular use. Use in other areas increases the likelihood that the plant is efficacious.

3. Mid level: If in addition to the ethnobotanical data, available phytochemical or pharmacological information is consistent with the use, this indicates a higher level of confidence that the plant may exert a physiological action on the patient.

4. High level: If both ethnobotanical and pharmacological data are consistent with the folk use of the plant, its use is classed in the highest level of validity and is considered efficacious.

## Results

In all, 20 plants were found to be used in treating racehorses in Trinidad and 97 were used in BC. Of these seven of the most evidently effective plants used in Trinidad and 33 of those used in BC are described and evaluated in this paper. In BC eighteen plants were used for wounds and abscesses and ten plants were used for anxiety and nervousness. The next largest group of plants (7) were used for hormone imbalances. This last category of treatment was not described in Trinidad. *Aloe vera*, *Pulmonaria officinalis *and *Medicago sativa *were reported to be used for exercise induced pulmonary haemorrhage. *Aloe vera*, *Curcuma longa *and *Ricinus communis *were used in both research areas but for different ailments.

### Ethnoveterinary remedies used in Trinidad

The ethnoveterinary usages of locally available plants for horses in Trinidad are summarised in Table 1. Twenty plants are used.

**Table 1:** Ethnoveterinary medicines used for horses in Trinidad and Tobago

Scientific name	Family	Common Name	Plant part used	Use
*Aloe vera*	Liliaceae	Aloes	Leaf gel	anhydrosis, Retained placenta, Tendon problems
*Capsicum annuum *L., *Capsicum frutescens*	Solanaceae	pepper	leaf	Anhydrosis
*Cecropia peltata*	Cecropiaceae	Bois canôt	Leaf	Anhydrosis, Kidney problems
*Cordia curassavica*	Boraginaceae	Black sage	Leafy branch	Grooming
*Curcuma longa*	Zingiberaceae	Turmeric	Rhizome	Retained placenta
*Desmodium *sp.	Fabaceae	Speedweed		Enhance performance
*Momordica charantia*	Cucurbitaceae	Caraaili	Vine	Tonic, blood purifier, skin rashes
*Mucuna pruriens*	Fabaceae	Cow itch	Leafy branch	Enhance performance
*Musa *species	Musaceae	Banana	Fruit	Diarrhoea
*Nasturtium officinale*	Brassicaceae	Watercress	Leaf	Increase blood count
*Nopalea cochenillifera*	Cactaceae	Rachette	Joint	Diaphoretic, tendon problems
*Oxalis corniculata*	Oxalidaceae	Speedweed		Enhance performance
Panicum maximum*	Poaceae	Wiz/Guinea grass	Leaf	Grooming
*Pimenta racemosa*	Myrtaceae	Bay leaves	Leaf	Diaphoretic
*Psidium guajava*	Myrtaceae	Guava	Leaf, bud	Diarrhoea
*Pueraria phaseoloides*	Fabaceae	Kudzu	Leaf	High protein feed
*Ricinus communis*	Euphorbiaceae	Castor bean leaf	Leaf	Tendon problems
*Stachytarpheta jamaicensis*	Verbenaceae	Vervine	Leaf	High protein feed

*Respondent identification was not confirmed.

#### Plants used for diarrhoea

Guava (*Psidium guajava*) leaves, young fruits and/or buds were boiled and mixed with mash or bran or a combination of both and given to the horse to eat by three respondents after orthodox treatments had been tried. One respondent used young green fruit of the banana (*Musa *sp.) including skins once for one horse. The banana fruit was boiled, crushed and mixed with the mash and this was given to the horse to eat. Another respondent used carrots (*Daucus carota*) (eight kg). One respondent reported a one-time use of stale cow dung, which was pushed down the horse's throat in order to obtain beneficial bacteria. This practice of using cow dung was confirmed by another respondent.

#### Plants used for tendonitis

Medicinal plants for tendonitis were preferred by those who believe that horses don't have much circulation from the knee down; therefore ice is seen to be of no value for swelling. One respondent claimed that treatment was based on the stage of injury. He believed that the herbal remedies were more effective in the first stages of injury and stressed that rest was the most important factor for the recovery process.

Tendon and ligament problems were described as the second biggest affliction after respiratory problems. Horses with sprained tendons or ligaments have joints of rachette (*Nopalea cochenillifera*) applied directly to the injured area. The mucilage obtained from inside the rachette joints may be mixed with flour and or Epsom salts. Two respondents practiced tendon splitting, or splitting of the affected suspensory ligament and the flexor tendon into the normal tissue above and below the lesion. Respondents do this to increase circulation to the affected area and thus enhance the healing process. Castor bean leaves (*Ricinus communis*) were quickly passed over a flame, and wrapped around the clay already placed on the injured tendon, which was then left to heal. Joints of rachette (*Nopalea cochenillifera*) were split open, mixed with aloes (*Aloe vera*) or clay, and packed on to the tendon. This poultice was said to help with the healing process and to keep "heat" from the damaged tissue or injured joint out of the tendon. Alternatively, leaves of wonder of world (*Kalanchoe pinnata*) were used to remove the "heat" from the injured leg. Wonder of world is claimed to have antiinflammatory properties. The rest of the treatment consists of rest and those trainers who believe that using ice has value use an ice pack to completely cover the leg.

Three interviewees blister flexor tendons or suspensory ligaments to help the healing process. The method consists of rubbing the tendon with iodine or mercuric iodine on a toothbrush for three days. This practice is stopped for three days and then another cycle is started. After the raw scab comes off, aloes (*Aloe vera*) is applied to help the tissues and skin heal. Blistering agents' remove the hairs from the injured part, there is localised swelling, the skin sloughs off and subcutaneous necrosis can also occur. Blistering necessitates rest since a long healing period is required. Horses were not blistered above the knee. The iodine is said to act as a counter irritant, which brings blood to damaged part, and the increased circulation enhances the healing process.

Bucked shins were described as an injury in the forelimb of young horses after exercise and were also blistered. There is periostitis of the plantar surface of the third metacarpal (or metatarsal) bone. Horses with tendon injuries were also taken to the sea for exercise to take the weight off the legs. Alternatively the injured leg is placed in brine from salted pigtails; both practices were said to harden the tendon. This remedy is thought to be over 30 years old. Aloes (*Aloe vera*) was also used for soreness in horses' joints, the gel is made into a paste, applied and then the joint is bandaged.

Poultices were made with river clay or white clay. Some buy the clay already prepared while others do their own preparation. Other poultices were made with a combination of clay, washing soap (hard bar) and glycerine and Epsom salts. The clay keeps the horses legs cool. Poultices were sometimes made with a combination of aloes, rachette, glycerine and Epsom salts, and were said to have a "drawing" effect.

Young castor bean leaves (*Ricinus communis*) or two to three young almond leaves were warmed and the veins were crushed (n.b. Trinidad almond is *Terminalia catappa*, this plant was identified from the literature). These leaves were put on minor injuries and bandaged. It is said that oil runs out of *Ricinus communis *leaves and cools the "heat" or swelling in the leg. Horses with bad tendon injuries were treated with rachette and aloes. This particular treatment is called "sweating it down." The plants were grated and packed on the leg. In terms of dosages all respondents used sufficient plant material to cover the area being treated. The leg is then wrapped with a football sock that has had the toe cut off. The sock is then tied at the bottom. The plants were thus packed inside the sock. The sock is then wrapped with a bandage to keep it in place. An alternative treatment is to put aloes on first, then wrap a heated bois canôt (*Cecropia peltata*) leaf on the leg, which is then bandaged with cotton. This practice is repeated for a few months. Trainers also rub a decoction of bay leaves (*Pimenta racemosa*), indigo blue and a scent like lavender (owner preference for scent) on their horses' sore muscles and quarters.

#### Plants used for grooming

Wiz is the horse racing term for a ball of dried plant material used for grooming. A wiz may be made up of wild carailli leaves (*Momordica charantia*) elephant or guinea grass (*Panicum maximum*) or wild senna leaves (*Senna alata*). A bundle of this dry grass (the plant tops) was beaten on a wall and stripped thin. It was then rolled into a ball and placed in the sun to dry. A wiz was best if left to age. This matted bundle was then rubbed on the horses' skin and was said to make the skin shiny. A wiz was used only on a clean horse. A bundle of branch tips of black sage (*Cordia curassavica*) (also called shining bush in the horse racing industry) was used before horses race to make the horse's coat shiny, as a coat cleaner and to remove the superficial dust. The dust from the horse's skin turns the bunch of leaves brown. A wet horse may be rubbed with wild carailli or wild senna leaves to cool them. Coconut oil (*Cocos nucifera*) was also used to make the coat shine. One respondent used carailli to treat rashes. The carailli vine was boiled and the water was then used to sponge the horse.

#### Plants used for hoof problems and other injuries

Wonder of the world (*Kalanchoe pinnata*), young banana leaves (*Musa *species), or castor bean leaves (*Ricinus communis*) were rolled with a bottle to burst the plant veins. The leaves were then passed quickly over a flame to warm them. Soft candle (whale fat) and Epsom salts were pasted on and the leaves were then placed on top. The whole thing was then wrapped with vet wrap or Elastoplast^®^. Alternatively turmeric root (*Curcuma longa*) was pounded and used. The entire foot was then placed in a bag or bandaged for three or four days and "sweated" for as long as it took to draw the inflammation out. This practice was used to draw infections out of injuries such as bruises from stones below the hoof. For cuts, aloes (*Aloe vera*) was bandaged on for two to three days.

#### Plants used as anthelmintics

Worm grass (*Chenopodium ambrosioides*) was used as an anthelmintic, but less so than in the past. The very infrequently used leucaena (*Leucaena leucocephala*) was said to make hairs from the horses' tail drop off.

#### Plants used for enhanced performance

Horse's hind quarters were occasionally rubbed with cow itch (*Mucuna pruriens*), this was said to help them come out of the boxes faster, since the plant acts as an irritant. Bay leaf (*Pimenta racemosa*) was used to bathe horses on race day, this was said to carry heat into body, which makes them run faster to get away from the sun's heat. Two plants called speedweed (*Oxalis corniculata *and *Desmodium *sp.) were used to enhance performance. The plants were fed to horses with the rest of their feed, not given specifically before a race.

#### Plants used for anhydrosis

If the horse did not seem to be sweating, or was dry coated, *Aloe vera *or two bois canôt leaves (*Cecropia peltata*) or grated rachette (*Nopalea cochenillifera*) was mixed with water and administered as a drench. Pepper leaves (*Capsicum annuum*, *Capsicum frutescens*) may also be used. It was thought that this "heats" the horse which makes it drink more water. These practices were said to "cool down" the horse's system and bring out the "heat", the animal sweats a few hours later. In previous times horses were taken to the river to stand up in the water for an hour after the race. Rachette (*Nopalea cochenillifera*) joints were pounded up, put in water, and given to horses to drink, they "sweat it out" and this helps them reduce their temperature. Horses were also bathed with bay leaves (*Pimenta racemosa*) to make them feel cool. A decoction of one or two cups of bay leaves was added to a half bucket of water, this liquid was then used to sponge the horse. Alternatively they were sponged with bay rum. Bay rum is a mixture of bay oil extracted from leaves of *Pimenta racemosa*, alcohol and water.

#### Plants used for retained placenta

Horses with retained placenta were seen to have a black discharge three days post partum. These horses were given a 7.5 cm piece of aloes (*Aloe vera*) each day for three days, and then purged with castor oil (*Ricinus communis*). One respondent used linseed oil mixed with aloe vera gel twice weekly. About half of a large leaf of *Aloe vera *was used. Another respondent used pounded turmeric rhizome (*Curcuma longa*). Turmeric was said to flush out the uterus. Horses were also given molasses water to drink, this was said to "clean them out." Horses were also given a combination of glycerine, Epsom salts and rachette (*Nopalea cochenillifera*) to treat inflammation.

#### Plants used for digestive problems

Aloes (*Aloe vera*) was boiled for five minutes and mixed with linseed oil. This was syringed down the horse's throat; some spit it out. Aloes was used for most internal problems and it was said to ease digestive problems. Subsequent to the administration of the *Aloe vera *the horse was given a purge with castor oil (*Ricinus communis*). Aloe leaves were also peeled and blended with water; this mixture was then combined with honey, and given orally with a syringe. A decoction of caraaili (*Momordica charantia*) vine was given orally as a digestive aid.

#### Plants used for bleeders – exercise induced pulmonary haemorrhage (EIPH)

Horses that collect blood in their lungs during or after a race were called "bleeders" (exercise induced pulmonary haemorrhage). To treat bleeders, honey and aloes were given orally. Sometimes the white of an egg was included. Additionally, pureed lemon juice was syringed into the horse's nostrils, this was said to curb bleeding by acting as an astringent. Watercress (*Nasturtium officinale*) was put in horses' food to "increase their blood count." Vervine (*Stachytarpheta jamaicensis*) and kudzu (*Pueraria phaseoloides*) plant tops were fed as high protein feeds.

#### Plants used for urinary problems

A decoction of bois canôt (*Cecropia peltata*) leaves was given as the drinking water. One respondent remembered seeing a veterinarian use the long stem of a pawpaw leaf (*Carica papaya*) as a catheter to clear a urinary blockage. To stimulate diuresis a decoction of the dry leaves of bois canôt (*Cecropia peltata*) was prepared in a bucket; a cup of this liquid in then put in the horses' drinking water. This was thought to assist in "cleaning out the bladder" of the horse.

#### Plants used for respiratory conditions

For bad head colds, horses were sweated or syringed with a cough medicine made of honey, garlic, and onion and boiled bois canôt leaves (*Cecropia peltata*). To "sweat" the horse, heated bricks from a dirt oven were put into a bucket with Vicks, peppermint oil or Foyles Balsam™. The horse's head was put in the bag, and the horse forced to inhale the steam.

### Ethnoveterinary remedies used in BC

The ethnoveterinary usages of locally available plants for horses in British Columbia are summarised in Table 2.

**Table 2:** Ethnoveterinary medicines used for horses in British Columbia

Scientific name	Family	Common name	Plant part used	Use
*Achillea millefolium*	Asteraceae	yarrow	dried aerial parts	fever
*Alchemilla vulgaris*	Rosaceae	lady's mantle	leaves	hormone imbalances
*Allium sativum*	Liliaceae	garlic	cloves	respiratory problems
*Aloe vera*	Liliaceae	aloe vera	leaf gel	hoof abscess, skin problems
*Althea officinalis*	Malvaceae	marshamallow	aerial parts	demulcent for devil's claw, prevent colic
*Althea *sp.	Malvaceae	mallow	aerial parts	counteract Lasix
*Arctium lappa*	Asteraceae	burdock	root	arthritis
*Arctostaphylos uva-ursi*	Ericaceae	uva-ursi	aerial parts	bladder infections
*Artemisia *sp.	Asteraceae	wormwood	aerial parts	endoparasites
*Astragalus membranaceous*	Fabaceae	astragalus	leaves	recovery
*Barosma betulina *and *B. crenulata*	Rutaceae	buchu	leaves	kidney tonic
*Berberis aquifolium*	Berberidaceae	Oregon grape	root	abscesses
*Calendula officinalis*	Asteraceae	calendula	infused flower oil & sulphur	front fetlock irritation
*Calendula officinalis*	Asteraceae	calendula	root	ringworm
*Calendula officinalis*	Asteraceae	calendula	flowers	eye problems, grass rash, sunburn, wounds
*Capsella bursa-pastoris*	Brassicaceae	shepherd's purse	aerial parts	skin rash, skin problems
*Capsicum *sp.	Solanaceae	cayenne	fruits	joint pain
*Cichorium intybus*	Asteraceae	chicory	aerial parts	stomach problems
*Coffee arabica*	Rubiaceae	coffee	roasted seeds	tonic
*Commiphora molmol*	Burseraceae	myrrh	resin, gum	abscesses, wounds
*Crataegus oxyacantha*	Rosaceae	hawthorn	berries, leaves or flowers	heart tonic
*Cucurbita pepo*	Cucurbitaceae	pumpkin	fruit flesh and seed	tapeworms
*Curcuma longa*	Zingiberaceae	turmeric	rhizome	arthritis
*Daucus carota*	Apiaceae	carrot	grated root	pinworms
*Echeveria elegans*	Crassulaceae	hen and chicks	leaves	hoof abscess
*Echinacea angustifolia*, *E. purpurea*, *E. pallida*	Asteraceae	echinacea	leaves and flowers	abscesses
*Equisetum arvense*	Equisetaceae	horsetail	aerial parts	arthritis
*Euphrasia officinalis*	Scrophulariaceae	eyebright	leaves	eye problems
*Filipendula ulmaria*	Rosaceae	meadowsweet	whole plant	arthritis
*Filipendula ulmaria*	Rosaceae	meadowsweet	dried aerial parts	Fever, blood thinner
*Fragaria virginiana*	Rosaceae	strawberry	leaf	hormone imbalances
*Galium *sp.	Rubiaceae	bedstraw	aerial parts	grass colic
*Glycyrrhiza glabra*	Fabaceae	licorice	root	arthritis, counteract Lasix, prevent colic, muscle soreness, synergy
*Harpagophytum procumbens*	Pedaliaceae	devil's claw	root	arthritis
*Humulus lupulus*	Cannabaceae	hops	strobiles	prevent colic, nerves, sedative
*Hypericum perforatum*	Hypericaceae	St John's Wort	flowers	abscesses
*Hyssopus officinalis*	Lamiaceae	hyssop	aerial parts	heart tonic
*Inula helenium*	Asteraceae	elecampane	aerial parts	abscesses, endoparasites, respiratory problems
*Laminaria *sp.*, Fucus *sp.	Laminariaceae, Fucaceae	kelp	leaf	abscesses
*Lavandula augustifolia*	Lamiaceae	lavender	aerial parts	soother
*Lavendula *sp.	Labiatae	lavender	flowers	anxiety, arthritis
*Leonorus cardiaca*	Lamiaceae	motherwort	aerial parts	wounds
*Magnolia acuminata*	Magnoliaceae	magnolia	leaves	anxiety
*Malva *sp	Malvaceae	mallow	aerial parts	respiratory problems
*Matricaria chamomilla*	Asteraceae	chamomile	aerial parts	eye problems
*Matricaria recutita*	Asteraceae	chamomile	flowers	prevent colic, hormone imbalances, muscle soreness, nervousness, stomach acid
*Medicago sativa*	Fabaceae	alfalfa	hay	bleeders
*Melaleuca alternifolia*	Myrtaceae	tea tree	oil	hoof abscess, wounds
*Mentha piperita*	Lamiaceae	peppermint	Leaves, aerial parts	intestinal problems, stomach acid
*Oenothera biennis*	Onagraceae	evening primrose	flowers	hormone imbalances
*Passiflora incarnata*	Passifloraceae	passion flower	aerial parts	hormone imbalances
*Passiflora incarnata*	Passifloraceae	passion flower	flowers	anxiety
*Petroselinum crispum*	Apiaceae	parsley	aerial parts	urinary cleanser
*Plantago major*	Plantaginaceae	broad-leaf plantain	leaf	abscesses, skin rashes
*Prunella vulgaris*	Lamiaceae	woundwort	aerial parts	wounds
*Pulmonaria officinalis*	Boraginaceae	lungwort	aerial parts	bleeders
*Ricinus communis*	Euphorbiaceae	castor bean	oil	abscesses
*Rubus idaeus*	Rosaceae	red raspberry	leaves	pregnancy
*Rubus ursinus*	Rosaceae	trailing wild blackberry	leaves	prevent colic
*Rumex crispus*	Polygonaceae	yellow dock	leaves	skin rash
*Salix alba*	Salicaceae	white willow	bark	arthritis, stomach lining, muscle soreness
*Salix alba*	Salicaceae	white willow	bark and/or leaf	fever
*Salvia *sp.	Lamiaceae	red sage	leaves	tonic
*Scrophularia nodosa*	Scrophulariaceae	figwort	aerial parts	wounds
*Scutellaria baicalensis*	Lamiaceae	baical skullcap	aerial parts, leaves	arthritis, skin rash
*Scutellaria lateriflora*	Lamiaceae	skullcap	aerial parts, leaves	revive gut flora, pain, nervousness
*Silybum marianum*	Asteraceae	Milk thistle	seed	arthritis, reduce blood pressure
*Stachys officinalis *synonyms *Stachys betonica*, *Betonica officinalis*	Lamiaceae	betony	aerial parts	wounds, prevent colic
*Stellaria media*	Caryophyllaceae	chickweed	aerial parts	skin problems
*Symphytum officinalis*	Boraginaceae	comfrey	root	counteract Lasix
*Symphytum officinalis*	Boraginaceae	comfrey	leaves	eye problems, abscess, hoof crack
*Symphytum officinalis*	Boraginaceae	comfrey	root	wounds
*Tanacetum parthenium*	Asteraceae	feverfew	dried aerial parts	fever
*Taraxacum officinale*	Asteraceae	common dandelion	aerial parts	intestinal problems, reduce blood pressure, straining
*Thymus *sp	Lamiaceae	thyme	leaves	coughs, colds
*Thymus *sp.	Lamiaceae	french thyme	aerial parts	endoparasites
*Tilia europea*	Tilaceae	linden	flowers	hormone imbalances
*Ulmus rubra, U fulva*	Ulmaceae	slippery elm	bark	soothe gut, abscesses, over-acidity, diarrhoea, wounds, gastroenteritis
*Urtica dioica*	Urticaceae	nettles	aerial parts	summer itch
*Valeriana officinalis*	Valerianaceae	valerian	root	nervousness
*Verbascum thapsus*	Scrophulariaceae	mullein	aerial parts	counteract Lasix, respiratory problems
*Viburnum opulus*	Caprifoliaceae	crampbark	bark	acute colic, cramps, respiratory problems, stomach ache
*Vitex agnus-castus*	Verbenaceae	agnus castus, chaste tree	berries	hormone imbalances
*Zanthoxylum americanum*	Rutaceae	prickly ash	bark	flush lactic acid from muscles, muscle soreness
*Zingiber officinalis*	Zingiberaceae	ginger	rhizome	intestinal problems

#### Plants used for abscesses and wounds

Slippery elm (*Ulmus rubra*, *U. fulva*) inner bark powder was placed on a plantain leaf (*Plantago major*), with the addition of kelp or powdered myrrh (*Commiphora molmol*) (without the resin). Hot castor oil (*Ricinus communis*) packs were also used for abscesses. An external treatment for abscesses consisted of a wash of comfrey tea (*Symphytum officinalis*). This tea could include an infusion of Oregon grape (*Mahonia aquifolium*).

A complementary internal treatment included equal parts of powdered Echinacea (*Echinacea angustifolia *or *Echinacea purpurea *or *Echinacea pallida*), (leaves and flowers) and elecampane (*Inula helenium*) (leaves and flowers), either mixed once a day with the food, or administered as a tea was added to the water for several weeks after completion of the external abscess treatment.

Tea tree (*Melaleuca alternifolia*) oil was used as a disinfectant (undiluted). Compresses were made of powdered aerial parts of: betony (*Stachys officinalis*), figwort (*Scrophularia nodosa*) and motherwort (*Leonorus cardiaca*). Comfrey (*Symphytum officinalis*) root was added. Equal amounts of the herbs were made into a paste with water, applied onto a gauze pad and placed onto the wound. Myrrh gum (*Commiphora myrrha*) was used for wounds. Woundwort (*Prunella vulgaris*) aerial parts were ground into a paste with calendula (*Calendula officinalis*) flowers. Three parts slippery elm (*Ulmus fulva*) bark powder was mixed with hot water and one part myrrh powder (*Commiphora myrrha*) and was given for pain. To stop the bleeding from a large cut or tear kitchen flour was applied, the wound was bandaged tightly then the horse was taken to the veterinarian. One or two leaves of comfrey were crushed and applied to cracks on the hoof and then bandaged. Undiluted tea tree oil was put directly on abscesses of the hoof and wrapped, or a pure commercial *Aloe vera *product was used.

#### Plants used for anxiety

Plants fed to alleviate anxiety in horses included leaves of magnolia (*Magnolia acuminata*) and (fresh or dried) flowers of passion flower (*Passiflora incarnata*). Alternatively, lavender tea (*Lavandula *sp.) or tincture was given in one bucket of water or placed on the feed. For nervousness and restlessness a handful of flowers of chamomile (*Matricaria recutita*, *Matricaria chamomilla*) or the content of a chamomile tea bag was added to the main meal. One or 2 tbsp valerian (*Valeriana officinalis*) ground root was given to a horse that froze in stressful situations. One tsp of combined equal amounts of powdered valerian, hops (*Humulus lupulus*) and skullcap (*Scutellaria lateriflora*) was put into the feed, twice a day. Valerian use was stopped 48 hours before a race so that it would not be present in the blood at race time.

#### Plants used for arthritis and sore joints

Powders of the following plants were added to the feed: turmeric (*Curcuma longa*); aerial parts of horsetail (*Equisetum arvense*) (silica content); aerial parts of baical skullcap (*Scutellaria baicalensis*) (inflammation, sedative) and lavender (*Lavandula *sp.) flowers. A tea made of licorice root (*Glycyrrhiza glabra*) (synergistic effect). Prickly ash bark/toothache tree (*Zanthoxylum americanum*) was reported to flush lactic acid and toxins from muscles. Milk thistle (*Silybum marianum*) seed and burdock root (*Arctium lappa*) were also used. Animals either self-medicated with white willow (*Salix alba*) or they were given white willow (*Salix *sp.) bark or meadowsweet (*Filipendula ulmaria*), aerial parts or root, for inflammation and pain. A combination of devil's claw (*Harpagophytum procumbens*) decoction and a demulcent such as marshmallow (*Althea officinalis*) (aerial parts), was put on the food.

#### Plants used for exercise induced pulmonary haemorrhage (EIPH)

One breeder used a commercial herbal product containing lungwort (*Pulmonaria officinalis*) compounds, bioflavonoids and vitamin K for EIPH. That breeder also used alfalfa hay [or soaked alfalfa pellets] in a 1 : 4 ratio with the regular hay. Furosemide, a diuretic often used in the treatment of EIPH, was thought to dehydrate the horse. To reduce this effect, a tea was given with 1 part each of the following: licorice (*Glycyrrhiza glabra*) root, aerial parts of mullein (*Verbascum thapsus*) or mallow (*Althea *sp.), and comfrey (*Symphytum officinalis*) root.

#### Plants used for endoparasites

Horses were dewormed four times a year with aerial parts of the following powdered herbs added to the feed daily for one week: Elecampane (*Inula helenium*), or wormwood (*Artemisia *sp.), cut finely or ground. Alternatively wormwood was given in equal combination with elecampane (*Inula helenium*) and thyme (*Thymus *sp.).

Occasional-use dewormers were french thyme (*Thymus *sp.), given 2 tbsp a day for week (1/2 the dose for a pony). Or one bucket of grated red carrot (*Daucus carota*) added to feed on a daily basis to reduce pinworms. To expel tapeworms 2–3 cups of chopped pumpkin flesh and seed (*Cucurbita pepo*) was added to the feed.

#### Plants used for eye problems, eye infections

An infusion with saline solution was made with equal parts of the following: eyebright (*Euphrasia officinalis*) fresh or dry leaves, calendula (*Calendula officinalis*) flowers, and comfrey (*Symphytum officinalis*) leaves. The infusion was strained carefully and used as an eyewash. The infusion was weakened as the condition improved. Eyebright (*Euphrasia officinalis*) (1 tbsp/day) was added to the food, with water, for under a week. Two tea bags of chamomile (*Matricaria chamomilla*) or 2 heaping tsps of fresh or dried chamomile herbs was steeped with 1 cup of hot water and strained before the liquid was used as an eyewash.

#### Plants used as a heart tonic

2 tbsp a day of hyssop (*Hyssopus officinalis*) paste was given in feed or 20 – 30 ml tincture was given in the drinking water to increase blood pressure. Berries, leaves or flowers of hawthorn (*Crataegus oxyacantha*) were said to be cardiotonic. Meadowsweet (*Filipendula ulmaria*) reportedly thinned the blood and removed pain. A paste was made of 2 tbsp dandelions (*Taraxacum officinale*) or milk thistle (*Silybum marianum*) and given in the feed to decrease blood pressure.

#### Plants used for hormone imbalances

For hormone imbalances a tea was made with one of the herbs given below or 1 tsp of the ground herb was put directly on the food. Leaves of strawberry (*Fragaria virginiana*), flowers of linden (*Tilia europea*) (safe for pregnant animals), flowers of evening primrose (*Oenothera biennis*) or flowers of chamomile (*Matricaria recutita *syn. *Matricaria chamomilla*) were used. Berries of agnus castus or chaste tree (*Vitex agnus-castus*) were utilised for severe cases. Chaste tree was said to stop production of testosterone (used as an herbal gelding). Leaves of lady's mantle (*Alchemilla vulgaris*) and aerial parts of passion flower (*Passiflora incarnata*) were also used. A tea of crampbark (*Viburnum opulus*) was given if the animal had cramps (cramps that the respondent thought were hormonally-linked).

#### Plants used during pregnancy

Dried leaves of red raspberry (*Rubus idaeus*) (1/4 cup) were mixed with one cup of water and put on top of the grain. This mixture was syringed into the horses' mouths if they did not eat it. It was used for the last month and a half of pregnancy.

#### Plants used for respiratory problems (snots)

Elecampane (*Inula helenium*) was mixed with crampbark, powdered or chopped root of liquorice and thyme (*Thymus *sp.) and was used for stable cough. Alternatively blended cloves of garlic (*Allium sativum*) were added to the feed. Crampbark powder (*Viburnum opulus*) was added to the feed of wind-broken horses.

One cup each of the following plants were blended and used as a hot mash in feed or as a tea for snots: elecampane (*Inula helium*), licorice (*Glycyrrhiza glabra*), thyme (*Thymus *sp.) (1/4 cup) and mullein. Cloves of garlic and fenugreek seeds (*Trigonella foenum-graecum*) were also added. In addition, pure garlic powder and mullein (*Verbascum thapsus*) were fed with grain (once or twice a day) until the horse's nose stopped running. Equal parts of white willow (*Salix alba*) bark and/or leaf; and dried aerial parts of each of the following were mixed together into a paste and given to feverish horses: feverfew (*Tanacetum parthenium*), meadowsweet (*Filipendula ulmaria*) and yarrow (*Achillea millefolium*).

#### Plants used for sore muscles, sprains, joint pain or reaction of horses to selenium shot in the chest

One heaping tsp of cayenne pepper (*Capsicum *sp.) was mixed with enough olive oil to make a paste which was then rubbed on the affected part. A purchased 1:5 cayenne tincture was substituted for the paste (if available).

#### Plants used for skin problems

An infusion of 1.5 tsp aerial parts of shepherd's purse (*Capsella bursa-pastoris*) steeped in 1.5 cups of water, was strained and used as a wash. Chickweed (*Stellaria media*) rinse or salve was applied to the affected area twice daily. Powdered sulphur was added to calendula (*Calendula officinalis*) infused oil and used for front fetlock irritation. External applications used for hypersensitivity reactions due to fly bites and other causes of skin irritation consisted of dried, crushed plantain (*Plantago major*) leaves and witchhazel (*Hamamelis virginiana*) added to rubbing alcohol and applied topically. A sting from a nettle plant (*Urtica dioica*) was soothed with fresh crushed shepherd's purse (*Capsella bursa-pastoris*) and/or yellow dock (*Rumex crispus*) leaves applied topically. An internal treatment consisted of a tea of dried baical skullcap (*Scutellaria baicalensis*) given in the feed.

One application of old car oil, or fish or cod liver oil was used topically to treat ringworm. The crushed root of calendula (*Calendula officinalis*) was then applied as a poultice to stimulate hair follicle growth three days later. Alternatively fluoride toothpaste was put on the affected areas and brushed off the following morning; this treatment was repeated until the problem resolved. Plants used for summer itch and sunburn included dried nettles (*Urtica dioica*) added to the feed. Calendula lotion or *Aloe vera *was used for grass rash and sunburn. Lastly, an infusion of aerial parts of shepherd's purse (*Capsella bursa-pastoris*) was used to wash the affected area.

#### Plants used to treat various intestinal conditions

For digestive problems one bottle of Guinness^® ^(beer) was administered orally or mixed in with the feed. Ginger (*Zingiber officinalis*) (powdered, liquid or crystallized) or chopped leaves of peppermint (*Mentha piperita*) was also administered orally or mixed in with food or water. Horses were allowed to self-medicate with organic dandelions (*Taraxacum officinale*).

#### Plants used for colic

Chamomile (*Matricaria recutita*) and peppermint (*Mentha piperita*) were used for stomach acid. White willow bark (*Salix *sp.) was used to repair the stomach lining. Slippery elm bark powder (*Ulmus fulva*) was used for over-acidity, diarrhoea and gastroenteritis. Bedstraw (*Galium *sp.) was used for grass colic – 1 handful of crumpled aerial parts in the feed. Skullcap (*Scutellaria lateriflora*) was given for pain. Licorice root (*Glycyrrhiza glabra*) was used for its synergistic action. For acute colic a crampbark (*Viburnum opulus*) paste was administered orally as a first aid measure before calling the veterinarian. Or a 1:1 mixture of skullcap and slippery elm bark powder and (1:4) licorice was given. Aerial parts of skullcap (*Scutellaria lateriflora*) were put in the feed for two to three days after the colic occurred to revive gut flora. One tbsp (15 ml) nutritional yeast was added to the feed everyday for prevention of colic.

#### Plants used to treat stress

The following herbs were used preventively before stressful situations: aerial parts of dry or fresh betony (*Stachys officinalis*); powdered hops strobiles (*Humulus lupulus*), was added to the feed daily or made into a tea; or powdered aerial parts of marshmallow (*Althea officinalis*). Powdered licorice root (*Glycyrrhiza glabra*), was given daily in advance of stressful situations. Slippery elm (*Ulmus fulva*) bark powder was given to soothe the gut. Chamomile (*Matricaria recutita*) flowers were recommended for high-strung horses. Fresh or dry leaves of wild blackberry (trailing wild blackberry, *Rubus ursinus*), were fed *ad lib*. The following were used as teas or as powders in the feed with chicory (*Cichorium intybus*), slippery elm bark powder and crampbark as the main ingredients. They were used separately or in combination. If used separately, 1 tbsp of each ingredient was used with yoghurt as a paste base. In combination, 1 tbsp of each herb was steeped in boiling water and 1 cup of the tea given to the horse in the drinking water or put in the feed: crampbark (*Viburnum opulus*) for stomach ache;

slippery elm bark powder (*Ulmus fulva*) for over-acidity, diarrhoea and gastroenteritis;

hops buds (*Humulus lupulus*) act as a sedative; chamomile (*Matricaria recutita*) and peppermint to soothe stomach acid; less peppermint (*Mentha piperita*) is used in a blend than if given alone; chicory (*Cichorium intybus*); white willow bark (*Salix *sp.) rebuilds stomach lining; skullcap (*Scutellaria lateriflora*) for pain and a nerve tonic; licorice root (*Glycyrrhiza glabra*) synergistic action.

#### Plants used as a tonic after races

Red sage (*Salvia officinalis*) tea (1 tbsp of leaves per cup of boiling water) was cooled and put into their mash. Bran mash with 1 cup brewed coffee was used after the race and at least two days before the next one. *Astragalus membranaceous *was used to help recovery from a long illness; 1 tsp to 1 tbsp was added to the feed. Lavender (*Lavandula augustifolia*) was hung upside down in the stable where the horse could not reach it; the smell was soothing.

#### Plants used for urinary problems including edema ("stocked-up")

Ten buchu leaves (*Barosma betulina *or *Barosma crenulata*), or uva-ursi leaves (*Arctostaphylos uva-ursi*), were fed to horses after races as a kidney tonic. For minor bladder infections powdered uva-ursi aerial parts and chopped or powdered leaves of dandelions (*Taraxacum officinale*), were mixed and fed every day until the horse's legs were no longer swollen, or the horse was no longer straining to urinate (usually one to three days). Either fresh or dried parsley (*Petroselinum crispum*) was added to the feed once a day or more often until the urine cleared up. Dandelion aerial parts were fed *ad lib*.

### Review of the ethnomedicinal literature

The review below (Table 3) describes a selection of the clinical trials and experimental studies using ethnopharmacologically accepted models that have verified the traditional and therefore ethnoveterinary use of the plants described in the results section. In the few cases in which clinical trials have not yet been carried out, the range of therapeutically important and relevant biological properties of the plant is provided. Recent research has indicated that *Betonica *and *Stachys *may be separate genera or subgenera and this should be taken into consideration when reviewing the pharmacological literature on betony [19].

**Table 3:** Non-experimental validation of ethnoveterinary remedies used for horses in BC and Trinidad

Species	Phytochemical and pharmacological information	References
*Arctostaphylos uva-ursi*	Leaves contain arbutin which is converted in alkaline urine to hydroquinone (antibacterial and anti-inflammatory action).	20–25
*Astragalus membranaceus*	*Astragalus *increases T-cell-mediated immune functions *in vitro*, in mice, and in uncontrolled trials in humans. Polysaccharide fractions enhance phagocytosis, increase macrophage numbers, and enhance humoral immunity. Astragalus root increases the immune-stimulating effects of interleukin-2 and acyclovir.	26–31
*Calendula officinalis*	*Calendula *is anti-inflammatory and promotes epithalization; it is also used for focal skin irritation.	32–34, 28
*Cecropia pachystachya*	*In vivo *studies of *Cecropia pachystachya *showed weak broncodilator activity and cardiovascular toxicity on endovenous administration on dogs and rabbits. *Cecropia obtusifolia *has shown antihypertensive, diuretic, hypoglycemic, analgesic and central depressor effects. An infusion prepared with the leaves of *C. obtusifolia *produced beneficial effects on carbohydrate and lipid metabolisms when it was administered to patients with type 2 diabetes.	35–36
*Cichorium intybus*	Four of six rat stomachs were protected from EtOH damage by aqueous extracts of *Cichorium intybus*.	37
*Cordia curassavica*	*Cordia curassavica *hexane extracts showed antibacterial activity against Gram-positive and Gram-negative bacteria. The crude dichloromethane extract of *Cordia curassavica *showed significant antiedematogenic activity and antinociceptive activity.	38–39
*Crataegus oxycantha*	Hawthorn (*Crataegus oxycantha*) may increase myocardial contractility and reduce peripheral vascular resistance. The *Crataegus *cohort in one study showed less marked symptoms of heart failure after 2 years (fatigue, stress dyspnoea, palpitations).	40–42
*Curcuma longa*	In a randomised, double-blind, placebo-controlled, parallel group clinical trial of P54FP, 61 client-owned dogs with osteoarthritis were randomly allocated to receive P54FP (an extract of *Curcuma domestica *and *Curcuma xanthorrhiza*) or a placebo orally twice daily for eight weeks. There was a statistically significant treatment effect in favour of P54FP (P = 0.012). The clinical efficacy of a formulation containing roots of *Withania somnifera*, the stem of *Boswellia serrata*, rhizomes of *Curcuma longa *and a zinc-complex (Articulin-F), was evaluated in a randomized, double-blind, placebo controlled, cross-over study in 42 patients with osteoarthritis for three months. Treatment with the herbomineral formulation produced a significant drop in severity of pain (P < 0.001) and disability score (P < 0.05).	43–44
*Desmodium adscendens*	The butanolic extract of *Desmodium adscendens *inhibits contraction of the ileum and trachea in guinea pigs. Three active triterpenoid glycosides were found. An extract of *Desmodium grahami *produced a concentration-dependent inhibition of spontaneous ileum contractions. The extract showed antimicrobial activity against pathogenic enterobacteria supporting its ethnomedical use for gastrointestinal disorders. Three antimicrobial isoflavones were isolated from *Desmodium canum*.	45–48
*Echinacea purpurea*	*Echinacea purpurea *has been investigated for its potential to activate the innate immune response. A time course study, using the time of sheep red blood cells (SRBC) immunization to mimic the onset of illness, examined the effects of 8 and 4 days of *Echinacea purpurea *treatment at 0.6 mL/kg/day. Only in the 4-day administration, with dosing beginning 1 hour after SRBC immunization, was there an observed enhancement of the antibody forming cell response. This supports the acute use of *Echinacea purpurea *in traditional medicine, and demonstrates the potential for enhancement of humoral and innate immune responses.	49
*Equisetum arvense*	*Equisetum arvense *has demonstrated hypoglycaemic and diuretic activity. The hydroalchoholic extract of stems of *Equisetum arvense *produced an antinociceptive effect and anti-inflammatory activity linked to beta-sitosterol, campesterol and isofucosterol. A standardized extract from horsetail (*Equisetum arvense*) was administered to 11 volunteers following a flavonoid-free diet for 8 days. Hippuric acid, the glycine conjugate of benzoic acid, increased twofold after drug intake.	50–51
*Euphrasia officinalis*	Eyebright (*Euphrasia officinalis*) has anti-inflammatory activity. Eyebright contains quercetin, a bioflavonoid that may inhibit mast cell degranulation.	52, 28
*Filipendula ulmaria*	Meadowsweet (*Filipendula ulmaria*) contains a heparin-like anticoagulant in the flowers. An ointment composed of flowers of *Filipendula ulmaria *was studied for its efficacy against uterine cervical cancer in 48 patients. Positive responses were recorded in 32 patients (67%), including 25 cases (52%) of complete regression of dysplasia.	53–55, 56
*Galium aparine*	Asperuloside, an iridoid, is a mild laxative and has anti-inflammatory activity.	56
*Glycyrrhiza glabra*	This plant has been studied for its synergistic properties and its usefulness for respiratory conditions.	57–60
*Harpagophytum procumbens*	Chrubasik has conducted large trials with human patients using devil's claw and found that it relieved pain. *Harpagophytum procumbens *preparation was used to treat ten horses for degeneration of the proximal intertarsal, distal intertarsal and tarsometatarsal joints and found to be equivalent to the phenylbutazone control. Devil's claw has a protective action against arrhythmia.	61–66
*Hyssopus officinalis*	Antimicrobial activity of hyssop is linked to polysaccharides, essential oil, caffeic acid, tannins, and specifically (-)-cis- and (-)-trans-3-pinanones. Polysaccharides and crude extracts were active against HIV-type 1 and HIV-3 and non-toxic to uninfected cells. Extracts suppress hyperglycemia. The dried plant does not have the toxin pinocamphone.	68–69
*Lavandula angustifolia*	Extracts, fractions and essential oil of *Lavandula angustifolia *are reported to have CNS-depressant, anti-convulsive, sedative, anti-bacterial effects. Lavender (*Lavendula *sp.) has been used as a nocturnal sedative for elderly patients in the form of an air freshener. It has shown benefits in cancer care and stress. The calcium channel blocking activity of the aqueous-methanolic extract of *Lavandula stoechas *flowers (LS) may be responsible for the folk uses. At a dose of 600 mg/kg of LS, mice were calm and relaxed.	70–72
*Lavandula augustifolia*	Forty-two patients with advanced cancer were randomly allocated to receive weekly massages with lavender essential oil and an inert carrier oil (aromatherapy group), an inert carrier oil only (massage group) or no intervention (four week courses). Sleep scores improved significantly in both the massage and the combined massage (aromatherapy and massage) groups. There were also statistically significant reductions in depression scores in the massage group.	73
*Magnolia grandiflora*	A Chinese prescription containing *Magnolia *bark and ginger rhizome among others, is used to treat mental illnesses. Administration of this decoction and fluoxetine produced beneficial effects on rats subjected to chronic mild stress. *Magnolia grandiflora *contains magnolol and honokiol which exhibit a central nervous system effect and muscle relaxant activity (Bastidas et al., 1998). An improved elevated plus-maze test in mice revealed the anxiolytic potential of honokiol from *Magnolia officinalis *and *Magnolia obovata*.	74–76
*Matricaria recutita *syn. *Matricaria camomilla*	Apigenin is the sedative ingredient in chamomile. Aqueous 70% methanol extracts of *Chamomilla recutita *inhibited the growth of *Helicobacter pylori*, a Gram-negative bacteria responsible for chronic gastritis, peptic ulceration and gastric cancer. Treatment with *Angelica sinensis *and *Matricaria chamomilla *reduced hot flushes in menopausal women.	77–81
*Mentha piperita*	Peppermint (*Mentha piperita*) may reduce intestinal spasm and in one study, enhanced gastric emptying. Several studies of the efficacy of peppermint oil on irritable bowel syndrome showed that its activity was linked to the relaxation of intestinal smooth muscle. One study reported that peppermint odour had a positive effect on running speed.	82–85
*Momordica charantia*	*Momordica charantia *has many medicinal properties.	86
*Mucuna pruriens*	The spicular hairs of the pod of *Mucuna pruriens *penetrate skin causing intense irritation. Hairs contain 5-hydroxytryptamine (serotonin) and the itching produced by the hairs is due to the liberation of histamine in the epidermal layer of the skin.	87–89
*Musa paradisiaca*	*Musa sapientum *var. Cavendishii contains soluble and insoluble dietary fibre that contributes to its hypo-cholesterolaemic effect. Other studies found that dried unripe plantain banana (*Musa sapientum *L. var.*paradisiaca*) was anti-ulcerogenic. One study found that extracts of both raw *Musa sapientum *Linn. *Musa paradisiacal bananas *protected the rat stomach from indomethacin-induced injuries. The extract from *Musa sapientum *Linn. had a significant healing effect on acetic acid-induced ulcers.	90–93
*Nasturtium officinale*	Histamine release inhibitors (flavonols and megastigmanes) were found in watercress (*Nasturtium officinale*). Phenethyl isothiocyanate (PEITC) which is released upon chewing of watercress (*Nasturtium officinale*) is a chemoprotective agent.	94–97
*Nopalea cochenillifera*	More studies need to be conducted on this plant. An oral glucose tolerance test showed that stems of *Nopalea cochinellifera *raises blood glucose levels in mice.	98
*Oxalis corniculata*	Five *Oxalis *species including *Oxalis corniculata *have been used to treat skin infections and unspecified microbial infections. Dichloromethane extracts of *Oxalis erythrorhiza *showed activity against methicillin-resistant and methicillin-sensitive strains of *Staphylococcus aureus *as well as towards five dermatophytes. Embelin also inhibits the five dermatophytes.	99
*Passiflora incarnata*	In one non-randomized clinical observatory trial a combination product composed of valerian root and passion flower extracts was evaluated using 20 ambulatory patients (Dhawan et al., 2004). The plant combination reduced occipital region central hyperactivity after 2 weeks. The anxiety and depression self rating decreased for all patients. Many other studies, and adverse reactions, are summarised in this paper.	100
*Petroselinum crispum*	Parsley's diuretic effect was validated in rat experiments. Six rats offered an aqueous parsley seed extract to drink, eliminated a significantly larger volume of urine per 24 h (P < 0.001) as compared to when they were drinking water, but less than that observed with known diuretics amiloride and furosemide. The rats served as their own controls.	101
*Pimenta racemosa*	Antinociceptive and anti-inflammatory effect activity was found in the leaves of *Pimenta racemosa*.	102
*Plantago major*	*Plantago major *contains several compounds that aid in wound healing. The polysaccharide fraction from *P. major *protects against pneumococcal infection in mice when administered systemically, with prechallenge by stimulation of the innate immune system.	103–105
*Pulmonaria officinalis*	The anti-coagulant glycopeptide from *Pulmonaria officinalis *reduces the death rate of animals with exogenous thromboplastmenia. T-100 anticoagulants were isolated from the ammonia extract of *Pulmonaria mollissima*. The anticoagulants consist of a peptide and a glycopeptide which in nontoxic doses causes stable hypocoagulemia in animals.	106–107
*Prunella vulgaris*	The polysaccharide prunelline has immunomodulation effects and some constituents have antioxidative, anti-inflammatory, and moderate activity on Gram positive bacteria. Other polysaccharides have antiviral activity. The aqueous fraction of the plant inhibits anaphylactic shock, allergic reactions, protects rat erythrocytes against haemolysis and kidney and brain homogenates against lipid peroxidation.	108–109
*Psidium guajava*	Leaf extracts of *Psidium guajava *act as antidiarrhoeic agents by a triple pronounced antibacterial, antiamoebic and antispasmodic action (inhibition of intestinal motility).	111–113
*Ricinus communis*	The wounded leaf of *Ricinus communis *contained increased free fatty acids and diacylglycerol and decreased in phospholipids. Leaves of *Ricinus communis *are nematicidal.	114–115
*Salix *spp.	A standardized willow bark extract was examined in 127 outpatients with osteoarthritis and rheumatoid arthritis in 2 randomized, controlled, double-blind trials with follow up for 6 weeks. No statistical differences were found. Ethanolic Salix extract 1520L inhibits COX-2-mediated PGE2 release through compounds that were not salicin or salicylate. In a 4-week blinded trial, 210 patients with an exacerbation of chronic low back pain were randomly assigned to receive an oral willow bark extract with either 120 mg (low dose) or 240 mg (high dose) of salicin, or placebo, with tramadol as the sole rescue medication. The numbers of pain-free patients in the last week of treatment were 27 (39%) of 65 in the group receiving high-dose extract, 15 (21%) of 67 in the group receiving low-dose extract, and 4 (6%) of 59 in the placebo group (P <0.001). Significantly more patients in the placebo group required tramadol (P <0.001) for each week of the study.	116–117
*Salvia officinalis*	*Salvia officinalis *is reported to have anti-bacterial, fungistatic, virustatic, astringent, eupeptic, hypotensive, anti-spasmodic, central nervous system-depressant actions, anti-inflammatory and anti-hydrotic effects. Its water and alcohol extracts have anti-viral activity. The chloroform extracts of *Salvia officinalis *leaves, and the active compound ursolic acid, showed anti-inflammatory properties after topical application.	118
*Scrophularia nodosa*	Phenolic fractions of aerial parts of *Scrophularia frutescens *and *sambucifolia *showed potent antibacterial activity. Saikosaponins from *Scrophularia scorodonia *had *in vivo *anti-inflammatory effects.	119–120
*Silybum marianum*	Several studies have been found that milk thistle (*Silybum marianum*) has liver protectant properties.	121–122
*Stachys officinalis*	The hydroalcoholic extract of *Stachys lavandulifolia *showed anxiolytic effects with lower sedative activity than diazepam. Aqueous 70% methanol extracts of *Stachys alopecuros *inhibited the growth of *Helicobacter pylori*.	123–126
*Stachytarpheta jamaicensis*	After intraperitoneal administration of gradual aqueous doses obtained from *Stachytarpheta jamaicensis *leaves in rats the following effects were seen: a reduction of motor activity and the alarm reaction, ataxia, sedation, analgesia, anesthesia, ptosis, piloerection, head tremors and a significant reduction of body temperature followed by apnea and the death of the animals. Iridoid ipolamiide and the phenylpropanoid glycoside, verbascoside, were found. The crude protein level of *Stachytarpheta jamaicensis *is fairly high.	127–129
*Tanacetum parthenium*	Feverfew (*Tanacetum parthenium*) has antinociceptive and anti-inflammatory effects attributed to the parthenolide content in the leaves and flowers.	130–131
*Taraxacum officinale*	Teas composed of common dandelion root and aerial parts are licensed in Germany for the treatment of biliary disorders, digestive and gastrointestinal complaints, and to provoke diuresis.	131, 28
*Ulmus fulva*	Slippery elm (*Ulmus fulva*) is effective on its own as a demulcent in dogs with mild stomach ulcers.	28
*Verbascum thapsus*	Mullein (*Verbascum thapsus*) leaves and flowers have expectorant and demulcent properties (from mucilagionous constituents) which are used to treat respiratory problems such as bronchitis, dry coughs, whooping cough, tuberculosis, asthma, and hoarseness. Mullein is mildly diuretic and has a soothing and anti-inflammatory effect on the urinary tract, and acts as a mild sedative. Leaf extracts of *Verbascum thapsus *have shown antiviral, antibacterial and antifungal activity.	132–134
*Viburnum opulus*	Water-soluble polysaccharide fractions were isolated from the squeezed berries of *Viburnum opulus*. Some plant polysaccharides have immunostimulating activity: they enhance phagocytosis.	135–136
*Vitex agnus-castus*	Animal experiments have shown evidence of a dopaminergic effect of *Vitex agnus-castus*.	137–139

## Discussion and conclusion

There have been very few studies conducted on the use of herbs for horses. In one study on exercise induced pulmonary haemorrhage (EIPH) researchers evaluated two Chinese herbal formulas used in the USA to reduce EIPH (Yunnan Paiyao and Single Immortal). They used a randomized cross-over design with an exercise test in five Thoroughbred horses [140]. They found a statistically significant increase in time-to-fatigue after the treatment with Single Immortal, but no other result. The herbs used to treat EIPH in British Columbia are not found in the typical Chinese lung healing formula. Trinidad has a small Chinese population (> 1%) and no Chinese herbs (herbs used in a manner consistent with the principles of Chinese traditional medicine) were recorded [4].

The Santa Rosa track has a turf course that is not used as frequently as the sand course. In the wet season the sand course is described as "sloppy". These track conditions may have led the respondents in Trinidad to describe tendon problems as the second biggest problem after lung problems. There are some links between historically Amerindian treatments and EVM used for horses in Trinidad. For example clay was used by Native American groups to treat broken bones in horses and humans [141]. Like the Trinidad respondents, Native Americans used blistering agents as horse stimulants [142]. Lastly Amerindians (Pawnee Omaha and Ponca) fed the pounded bulbs of *Oxalis stricta *and *Oxalis violacea *to horses to make them fleet [143].

Participants in Trinidad were more reluctant to give specific dosages than their counterparts in BC. Several of the participants in Trinidad claimed that they previously used ethnoveterinary remedies but declined to specify what they had used in the past. The use of cow itch on race day is considered an offence by the Trinidad and Tobago Racing Authority; however there was no indication that the un-revealed plants mentioned above were also in this illicit category. Participants in BC also had an "illicit" plant: coffee was being used as a tonic after races – however a withdrawal period was observed.

Many of the plants being used for horses in BC were purchased as already formulated products. This fact reflects the different statuses of medicinal plant use in the two research areas. In Canada, there are several associations of alternative health practitioners and many certifying bodies. Canada also has a new Natural Health Products policy that regulates what is sold over the counter. It is difficult to compare the expenditure on horses in Trinidad and BC since statistics for Trinidad horses do not exist. However it is likely that more money is spent on medicinal products for horses in BC. A 1998 Canadian National Horse Industry Study showed that the total annual expenditure on grooming and health products was $90,000 or $105/horse/year [144].

Herbal medicine also has greater status in Canada because the plants of European-origin have been evaluated by the German Commission E or by Chinese scientists. Trinidad, in contrast, has one regional association of herbal practitioners – the Caribbean Association of Researchers and Herbal Practitioners (CARAPA), which was formed in 1998. This grouping consists largely of scientists, other professionals and only a few of the most prominent herbalists. Very few clinical trials have been conducted on plants that are native to the Caribbean. Most labelled and standardized products are foreign in origin. Rather than being available for purchase the Trinidad remedies listed in this paper were self-prepared by the users.

The largest category of plants used for horses in BC was for wounds and abscesses. The next largest category was for anxiety and nervousness. The third largest group was used for hormone imbalances. This last category of treatment was not described in Trinidad.

More research has been carried out on the temperate and Chinese plants used in BC and there is a greater commercial production of the plants being used for horses there. Therefore the BC ethnoveterinary remedies have stronger evidence of efficacy than those in Trinidad. This may also explain why there are more equine ethnoveterinary remedies that are used with greater confidence in BC than in Trinidad and Tobago. The tropical plants *Aloe vera *and *Curcuma longa*, two extensively researched plants, were being used in both areas. The ethnoveterinary use of *Ricinus communis *is similar to the ethnomedicinal use described in early British herbals [4]. These herbals later became global standard texts especially in those areas (like Trinidad and Canada) with a British colonial heritage. Some of the ethnoveterinary remedies used in Trinidad such as firing and blistering are no longer recommended in orthodox veterinary medicine, but these and some of the other ethnoveterinary remedies still used in Trinidad can be found in older Veterinary textbooks [145,146] and their use in Trinidad may originate from those sources.
